# Distribution of Fos-Like Immunoreactivity, Catecholaminergic and Serotoninergic Neurons Activated by the Laryngeal Chemoreflex in the Medulla Oblongata of Rats

**DOI:** 10.1371/journal.pone.0130822

**Published:** 2015-06-18

**Authors:** Xiaolu Wang, Ruichen Guo, Wenjing Zhao

**Affiliations:** 1 Institute of Clinical Pharmacology, Qilu Hospital of Shandong University, Jinan, China; 2 Key Laboratory of Cardiovascular Remodeling and Function Research, Qilu Hospital of Shandong University, Jinan, China; University of Leicester, UNITED KINGDOM

## Abstract

The laryngeal chemoreflex (LCR) induces apnea, glottis closure, bradycardia and hypertension in young and maturing mammals. We examined the distribution of medullary nuclei that are activated by the LCR and used immunofluorescent detection of Fos protein as a cellular marker for neuronal activation to establish that the medullary catecholaminergic and serotoninergic neurons participate in the modulation of the LCR. The LCR was elicited by the infusion of KCl-HCl solution into the laryngeal lumen of adult rats in the experimental group, whereas the control group received the same surgery but no infusion. In comparison, the number of regions of Fos-like immunoreactivity (FLI) that were activated by the LCR significantly increased in the nucleus of the solitary tract (NTS), the vestibular nuclear complex (VNC), the loose formation of the nucleus ambiguus (AmbL), the rostral ventral respiratory group (RVRG), the ventrolateral reticular complex (VLR), the pre-Bötzinger complex (PrBöt), the Bötzinger complex (Böt), the spinal trigeminal nucleus (SP5), and the raphe obscurus nucleus (ROb) bilaterally from the medulla oblongata. Furthermore, 12.71% of neurons with FLI in the dorsolateral part of the nucleus of the solitary tract (SolDL) showed tyrosine hydroxylase-immunoreactivity (TH-ir, catecholaminergic), and 70.87% of neurons with FLI in the ROb were serotoninergic. Our data demonstrated the distribution of medullary nuclei that were activated by the LCR, and further demonstrated that catecholaminergic neurons of the SolDL and serotoninergic neurons of the ROb were activated by the LCR, indicating the potential central pathway of the LCR.

## Introduction

The laryngeal chemoreflex (LCR) is a specific response initiated by chemical stimulation in the laryngeal lumen that results in series of reactions including respiratory inhibition, glottis closure, bradycardia and redistribution of blood flow [1,2]. This protective reflex is a primary function to prevent food and gastric juices from entering the lower respiratory tract [3,4]. However, severe apnea caused by prolonged LCR is considered one factor in sudden infant death syndrome (SIDS) [5–7].

Polymodal nociceptors on the mucosa of the laryngeal lumen receive and transmit the sensory information to the nucleus of the solitary tract (NTS) via the superior laryngeal nerve (SLN) [7]. The laryngeal motoneurons (LMN) located in the nucleus ambiguus [8–10] receive inputs from medullary nuclei including the area postrema (AP), NTS, rostral ventral respiratory group (RVRG), reticular formation, retroambiguus nucleus (RAmb), dorsal motor nucleus of the vagus, lateral tegmental field and raphe nuclei [11–17]. Fos protein is synthesized in neuronal nuclei in response to stimulation [18,19]. It has been reported that Fos-like immunoreactivity (FLI) is expressed in the rat hypothalamus and the amygdala of the forebrain after a chemical solution is infused into the rat laryngopharyngeal area [20]. However, it remains unknown which specific medullary nuclei are involved in the LCR.

Our previous study reported that the LMN receive multiple neurochemical inputs for the control of laryngeal behaviors and revealed the presence of both catecholaminergic and serotoninergic synaptic terminals in the caudal nucleus ambiguus [21]. Close appositions were identified between the LMN and tyrosine hydroxylase-immunoreactivity (TH-ir) as well as serotonin-ir terminals [22,23]. The nuclei showing overlap of catecholaminergic regions with laryngeal pre-motoneurons were the AP, NTS and the ventral lateral medulla [8–10,24]. The nuclei showing overlap of serotoninergic regions with laryngeal pre-motoneurons were the raphe nuclei [15,25,26]. Furthermore, studies of SIDS pathology have emphasized the functions of catecholamines [27–29] and the significance of the medullary serotonin pathway [30]. Therefore, it is assumed that the medullary catecholaminergic and serotoninergic neurons may play important roles in the control of the LCR.

Here, we aim to (1) localize and quantify the medullary nuclei activated by the LCR using immunofluorescence detection of FLI and (2) identify the medullary catecholaminergic and serotoninergic neurons activated by the LCR by using double labeling of TH-ir/serotonin-ir and FLI.

## Materials and Methods

### Animal preparation

Ten male Sprague Dawley rats (250–280 g) were included in the experiment. Rats were divided into two groups, five in the experimental group and five in the control group. The study protocol was approved by the Animal Ethics Committee of Qilu Hospital of Shandong University, Jinan, China (approval number: DWLL-2013-029). All animals were adaptively raised in a dark, quiet environment to avoid external stimuli.

After an overnight fasting, animals were anaesthetized with pentobarbital sodium solution (50 mg/kg, i.p.) following the administration of atropine (20 μg/kg, i.p.). Administration of additional pentobarbital sodium (3 mg/kg, i.p.) was given to maintain anesthesia during the surgery and LCR elicitation until sacrifice. Rectal temperature was maintained at 35–37°C with a homeothermic heating blanket. In the experimental group (n = 5), the rats were placed in a supine position and fixed on the operating table. After shaving and disinfecting the skin in the surgical area, a midline skin incision was cut, and tissues were bluntly separated to expose the larynx and the trachea. A cruciate incision was made in the sixth tracheal ring. A PE240 cannula was inserted rostrally to the laryngeal lumen as the infusion tube. A T-incision was cut in the laryngopharynx. Another PE240 cannula was inserted caudally to the laryngeal lumen as the drainage tube. A cavity of the larynx was made for receiving chemical stimulus. A midline skin incision was made to expose the xiphoid cartilage at the level of sternum. The xiphoid cartilage was separated and freed from the sternum but remained connected to the trunk musculature. The xiphoid cartilage was connected to the BL-420V Biological Data Acquisition & Analysis System (Chengdu TME Technology Co, Ltd. China) with a muscular tension transducer. Adjustment was made to record the muscle tension diagram (MTD) of the trunk musculature, showing the respiratory rhythm and intensity. Then, animals were left unconsciously for 4 h after surgery to minimize the Fos expression caused by surgery or any other uncertain factors. Control rats received the same surgery as experimental rats.

### LCR elicitation

Before stimulation, a baseline MTD of the trunk musculature was recorded. Experimental animals were subjected to the following chemical stimulation protocol: 2 mL of stimulus solution (0.5 M KCl-0.03 M HCl in distilled water) at room temperature was manually infused through the laryngeal lumen from the infusion tube and then drained from the drainage tube in 1 min. A 5 min pause was left between each infusion cycle. This sequence was performed 10 times over the course of 1 hour. At the same time, the trunk musculature tension signal was recorded. All rats were undisturbed with anesthesia for 1 h post-LCR elicitation to allow Fos expression to reach its maximum [20].

Although some innocuous solution like milk, saline and distilled water were used in the LCR elicitation [31–33], KCl-HCl was most effective in eliciting upper airway reflexes from laryngeal mucosa [20,34]. Rats in the control group were treated as in the experimental group except without KCl-HCl stimulation.

### Immunofluorescence

Animals were sacrificed and perfused transcardially with 300 mL saline, followed by 300 mL of 4% paraformaldehyde-0.01 M phosphate buffered saline (PBS, pH 7.4). Then, the medulla oblongata was removed immediately and post-fixed in the same fixative overnight at 4°C. The fixed medulla oblongata was dehydrated in 30% sucrose solution at 4°C for 24 h. Next, transverse sections (40 μm) were cut with a cryostat (Leica CM1950, Leica Microsystems Inc.). Serial sections were collected in five vials containing 0.01 M PBS in sequence. The interval of adjacent sections in each vial was 200 μm. Three vials of sections of each rat were required for the immunofluorescence assays. After incubation in 10% normal donkey serum (Jackson ImmunoResearch Laboratories Inc. Lot: 115072)-0.3% Triton X-100 (J &K Scientific Ltd. Lot: LLA0L39)-0.01 M PBS for 30 min, primary antibodies were added. Sections were incubated for 24 h with goat anti-c-fos (1:100, Thermo Fisher Scientific Inc. Lot: PA1-18329), along with rabbit anti-vesicular acetylcholine transporter (VAChT) (1:1000, Sigma-Aldrich, Lot: 122M4788V), rabbit anti-TH (1:500, Thermo Fisher Scientific Inc. Lot: PA1-18315) or rabbit anti-serotonin (1:4000, Sigma-Aldrich, Lot: 033M4805). Next, sections were incubated for 12 h in 5% normal donkey serum-0.3% Triton X-100-0.01 M PBS with FITC-conjugated donkey anti-rabbit IgG (1:200, Jackson ImmunoResearch Laboratories Inc. Lot: 114191) and TRITC-conjugated donkey anti-goat IgG (1:200, Jackson ImmunoResearch Laboratories Inc. Lot: 111778). All incubations were performed at 4°C with continuous gentle agitation and followed by 3 × 10 min washes in PBS. Eventually, sections were spread on the slides in rostrocaudal sequence, after which they were mounted with VECTASHIELD Mounting Medium (Vector Laboratories Inc. Lot: H-1000) and coverslipped.

### Data Analysis

Slides were observed with a fluorescence microscope (Olympus BX51, Olympus Corporation). To identify different levels of the serial sections, the obex was used as a point of reference and defined as the rostral end of the central canal (CC). We observed that the entire medulla oblongata ranged from 1.8 mm caudal to the obex to 3.0 mm rostral to the obex. Neurons double-labeled with VAChT-ir and FLI-ir in the caudal nucleus ambiguous were the LMN activated during the LCR. Double-labeling of TH-ir/serotonin-ir and FLI indicated catecholaminergic/serotoninergic neurons activated in the LCR. The immunofluorescent images were processed and analyzed with Image J, version 1.48. Atlas templates from The Rat Brain in Stereotaxic Coordinates (5th edition) by Paxinos and Watson were referred to identify the regions where the FLI was located in and to sum the number of FLI in each nuclei. The numbers of immunoreactive staining in each nucleus were counted bilaterally and totaled rostrocaudally. Data are presented as the mean ± SD. Statistics were performed using GraphPad Prism 5. Depending on the sample size, distribution and number, the two-sample t-test was used for the comparison of control and experimental conditions. Differences were considered statistically significant at *p* < 0.05.

## Results

### Respiratory disturbance during the LCR

**Fig 1:**
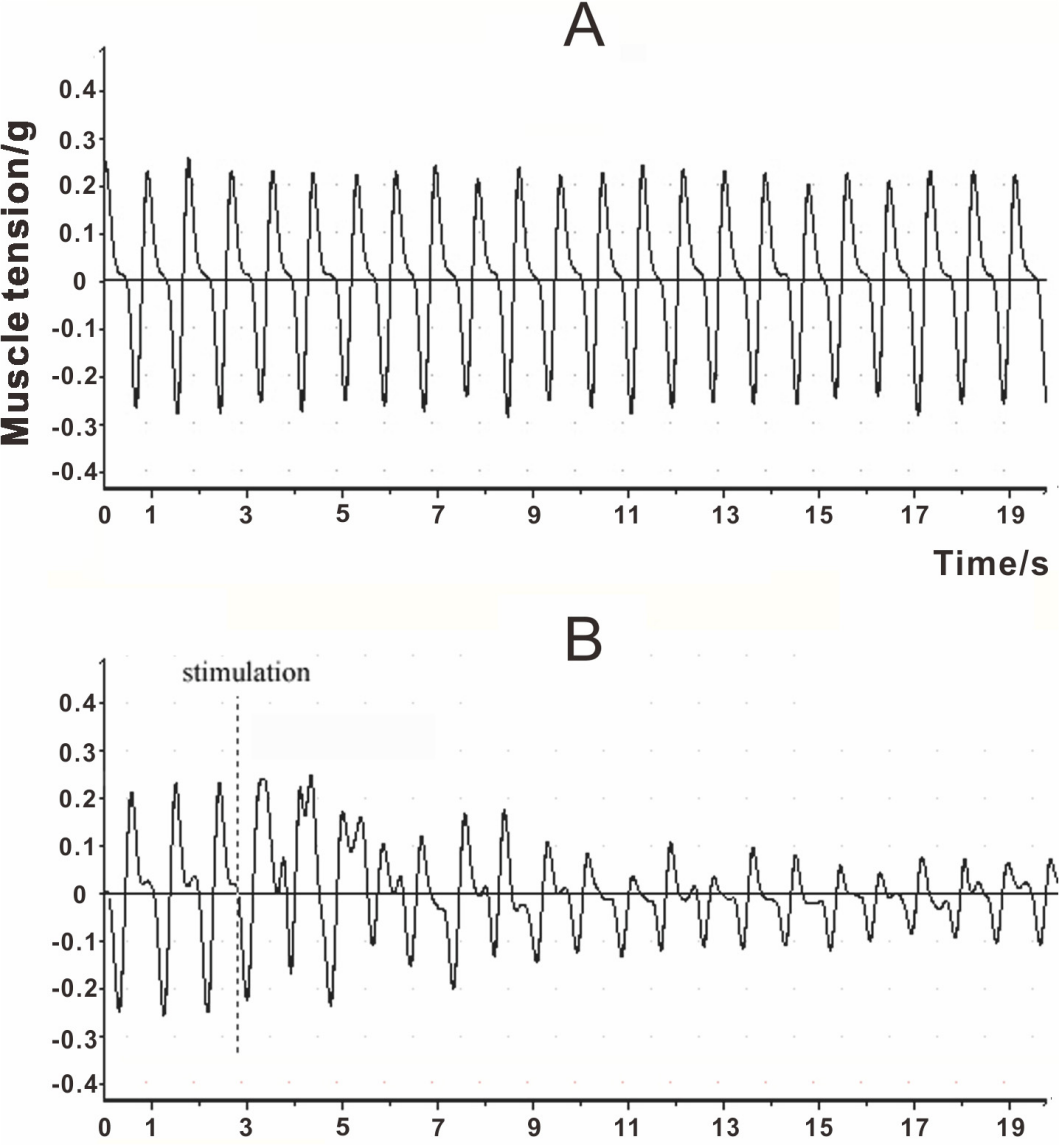
The muscle tension diagram (MTD) of trunk musculature acquired from a rat in the experimental group. (A) The baseline trunk musculature tension; (B) The interruption of trunk musculature tension during infusion of KCl-HCl solution in the rat laryngeal lumen at initial stimulation.

The baseline for trunk musculature tension is shown in Fig 1A, indicating the normal rhythm and frequency of respiration. With the infusion of KCl-HCl solution, respiration was disturbed immediately (Fig 1B). The amplitude of trunk musculature tension decreased by nearly 50% (from 0.2 g to 0.1g) and lasted until the end of single stimulation. Respiration recovered to baseline levels during resting intervals of 5 min. After four to five cycles of infusion, the respiratory disturbance caused by LCR was diminished. No animal exhibited apnea during stimulation.

### Distribution of FLI in the medulla

**Fig 2:**
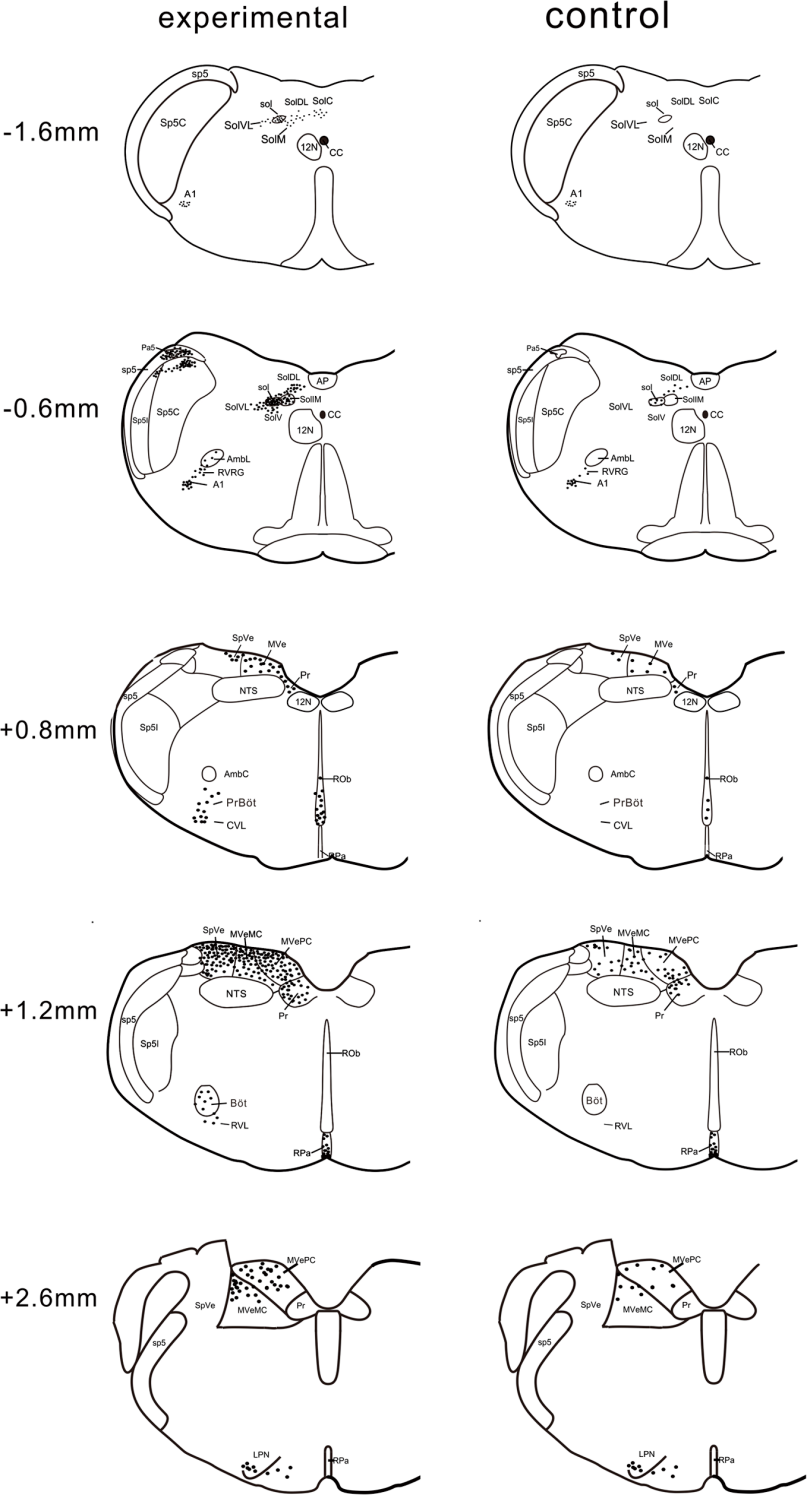
The distribution of FLI at the representative levels of the medulla from the control and experimental groups. 12N, hypoglossal nucleus; A1, A1 noradrenaline cells; AmbL, AmbC, loose, compact formation of ambiguus nucleus; AP, area postrema; Böt, Bötzinger complex; CC, central canal; CVL, caudal ventrolateral reticular nucleus; LPN, lateral paragigantocellular nucleus; MVePC, MVeMC, parvocellular and magnocellular parts of the medial vestibular nucleus; NTS, nucleus of the solitary tract; Pa5, paratrigeminal nucleus; PrBöt, pre-Bötzinger complex; Pr, prepositus nucleus; ROb, raphe obscurus nucleus; RPa, raphe pallidus nucleus; RVL, rostral ventrolateral reticular nucleus; RVRG, rostral ventral respiratory group; sol, SoLC, SolDL, SolVL, SolV, SoLM, SolIM, SolI, solitary tract, nucleus of the solitary tract, commissural, dorsolateral, ventrolateral, ventral, medial, intermediate and interstitial part; sp5, Sp5C, Sp5I, spinal trigeminal tract, caudal, interpolar part of spinal trigeminal nucleus; SpVe, spinal vestibular nucleus; VLR, ventrolateral reticular complex; VNC, vestibular nuclear complex.

FLI was observed in the nuclei of immunoreactive neurons as red staining of variable intensity. Fig 2 shows the distribution of FLI in representative levels of the medulla oblongata from the control and experimental groups. All the FLI was expressed bilaterally. The levels and bilateral counts of FLI in various nuclei are illustrated along the longitudinal axis of the medulla from the experimental and control groups (Table 1).

**Table 1:** The numbers and distribution of bilateral FLI in various medullary nuclei at the indicated longitudinal levels in the experimental and control groups.

Nucleus	experimental (n = 5)		control (n = 5)	
	level	Numbers (mean ± SD)	level	Numbers (mean ± SD)
**NTS**	−1.6 to +0.4	892 ± 136**	−0.8 to +0.2	225 ± 35
**VNC**	+0.6 to +3.0	4424 ± 105**	+0.6 to +3.0	2739 ± 68
**AmbL**	−0.6 to −0.2	17 ± 3**	-	-
**A1**	−1.6 to −0.2	246 ± 16	−1.6 to −0.2	219 ± 20
**RVRG**	−0.6 to obex	66 ± 5**	-	-
**VLR**	obex to +1.6	144 ± 38**	-	-
**PrBöt**	+0.8 to +1.0	25 ± 4**	-	-
**Böt**	+1.2 to +1.6	66 ± 15**	-	-
**LPN**	+2.2 to +3.0	190 ± 33	+2.2 to +3.0	177 ± 29
**SP5**	−1 to obex	775 ± 29**	-	-
**ROb**	obex to +1.2	191 ± 19**	obex to +1.2	58 ± 12
**RPa**	+1.0 to +2.0	95 ± 11	+1.0 to +2.0	89 ± 9
**Total**	1.6 to +3.0	7131 ± 711	-1.6 to +3.0	3447 ± 173

*p < 0.05

**p < 0.01, two-sample t-test, n = 5

As shown in Fig 2, in the control group, only a few FLI neurons were scattered in the NTS, including the solitary tract (sol), SolDL, and the commissural, interstitial, ventral, ventrolateral parts of the NTS (SolC, SolI, SolV, SolVL). Many more neurons showing FLI were distributed in the VNC, including the medial vestibular nucleus (MVe), the parvocellular and magnocellular parts of the MVe (MVePC, MVeMC), the spinal vestibular nucleus (SpVe) and the prepositus nucleus (Pr). In the ventral medulla, some of the FLI neurons were distributed among the A1 noradrenaline cells (A1) and lateral paragigantocellular nucleus (LPN). Several FLI neurons were observed in the ROb and the raphe pallidus nucleus (RPa).

**Fig 3:**
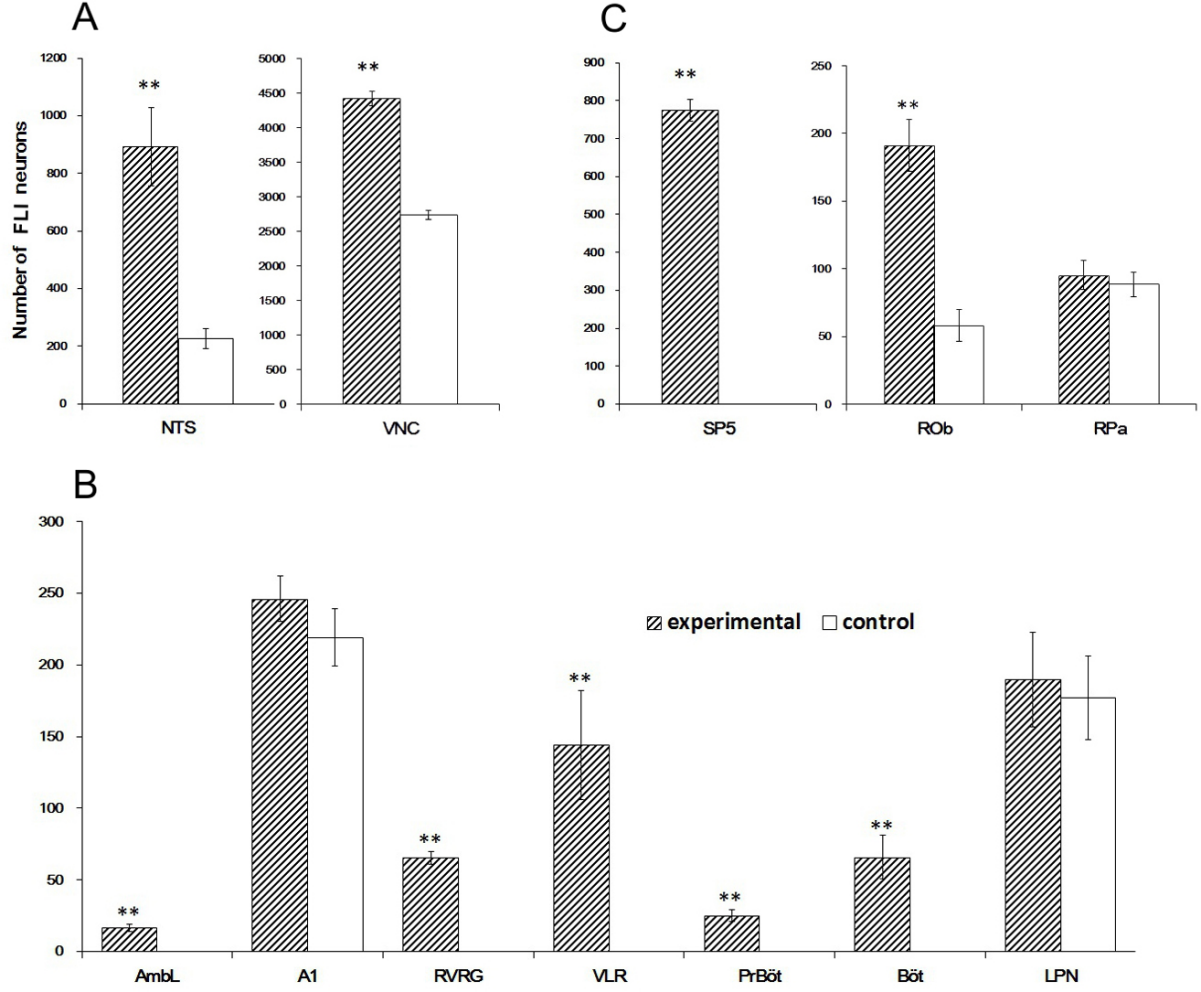
Comparison of bilateral FLI neurons between control and experimental groups in various rostrocaudal medullary nuclei in the dorsal medulla (A); the ventral medulla (B); and the other medullary regions (C). Abbreviations are defined in the legend of Fig 2.

In experimental rats, elicitation of LCR selectively promoted FLI in several nuclei. As shown in Figs 2 and 3, only the experimental group showed FLI in the medial and intermediate parts of the NTS (SolM, SolIM), AmbL, RVRG, PrBöt, Böt, VLR including caudal and rostral ventrolateral reticular nucleus (CVL, RVL) and SP5 including spinal trigeminal tract (sp5), the caudal and interpolar parts of the spinal trigeminal nucleus (Sp5C, Sp5I), and the paratrigeminal nucleus (Pa5). Compared with the control group, a region of FLI in the experimental group appeared to be qualitatively stronger in the NTS (sol, SolDL, SolI, SolV, SolVL, SolC), VNC (MVe, MVePC, MVeMC, SpVe, Pr) and ROb (two samples t-test, n = 5, *p* < 0.01). There was no significant difference in the FLI expression in A1, LPN and RPa between the control and experimental groups (two-sample t-test, n = 5, *p* > 0.05).

In summary, the FLI activated by LCR was distributed in the NTS (sol, SolDL, SolI, SolV, SolVL, SolIM, SolM, SolC), VNC (MVe, MVePC, MVeMC, SpVe, Pr), AmbL, RVRG, VLR (CVL, RVL), PrBöt, Böt, SP5 (Sp5C, Sp5I, Pa5, sp5), and ROb.

**Fig 4:**
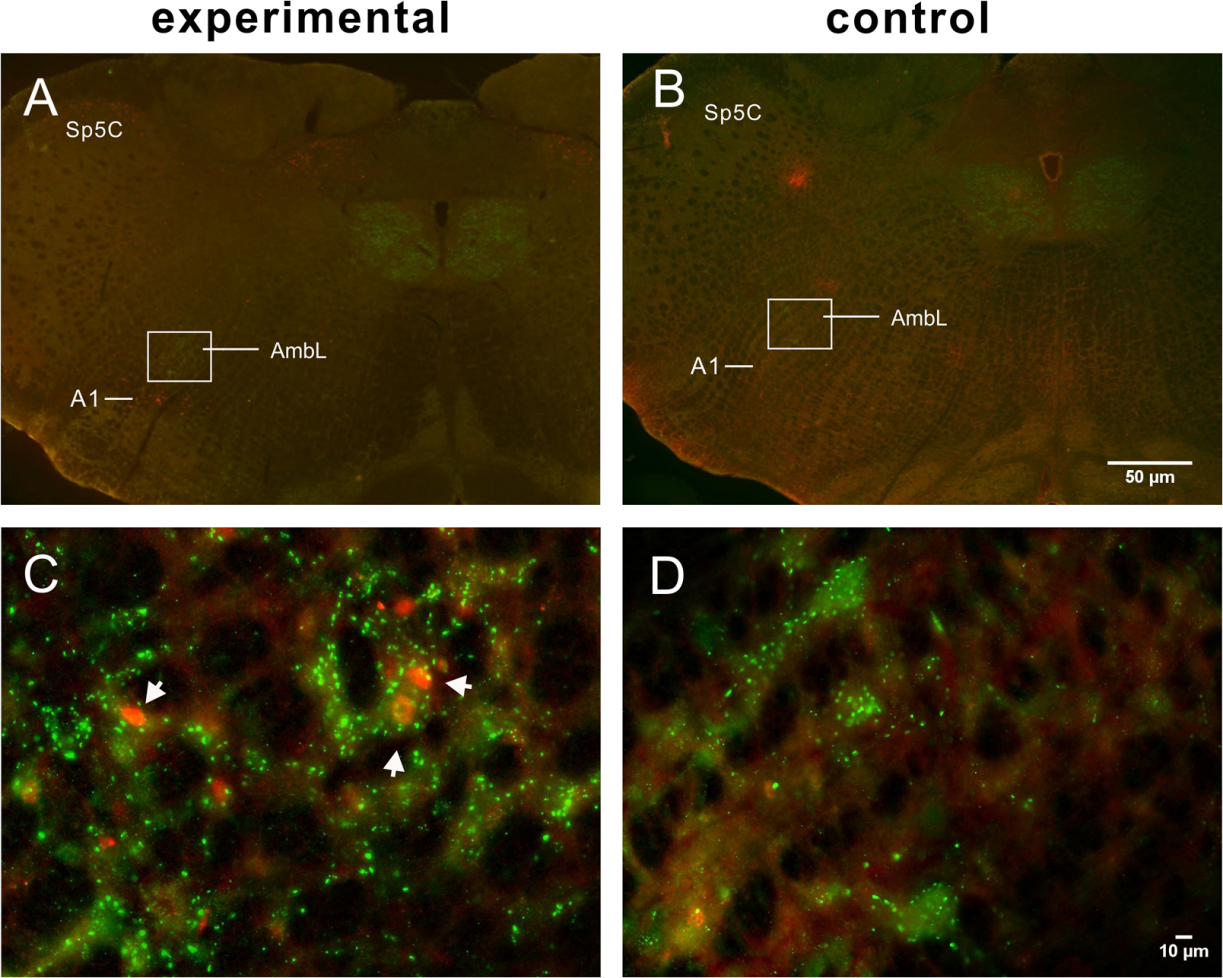
Low-power micrographs of the caudal medulla (0.6 mm caudal to the obex) showing dual-labeled FLI and VAChT-ir neurons from experimental (A) and control (B) rats. Boxed areas in A and B, indicating the AmbL, are enlarged in C and D, respectively, showing double-labeled neurons (white arrow) in the experimental rat but only VAChT-ir neurons in the control rat. Scale bar = 50 μm (applies to A, B), 10 μm (applies to C, D).

Sparse labeling was encountered in the AmbL (17 ± 3, n = 5) between 0.2 mm and 0.6 mm caudal to the obex (Fig 4). The FLI-positive neurons in the AmbL also showed VAChT immunoreactivity, indicating that the LMN are activated by LCR from the experimental group.

### Distribution of catecholaminergic FLI neurons

**Fig 5:**
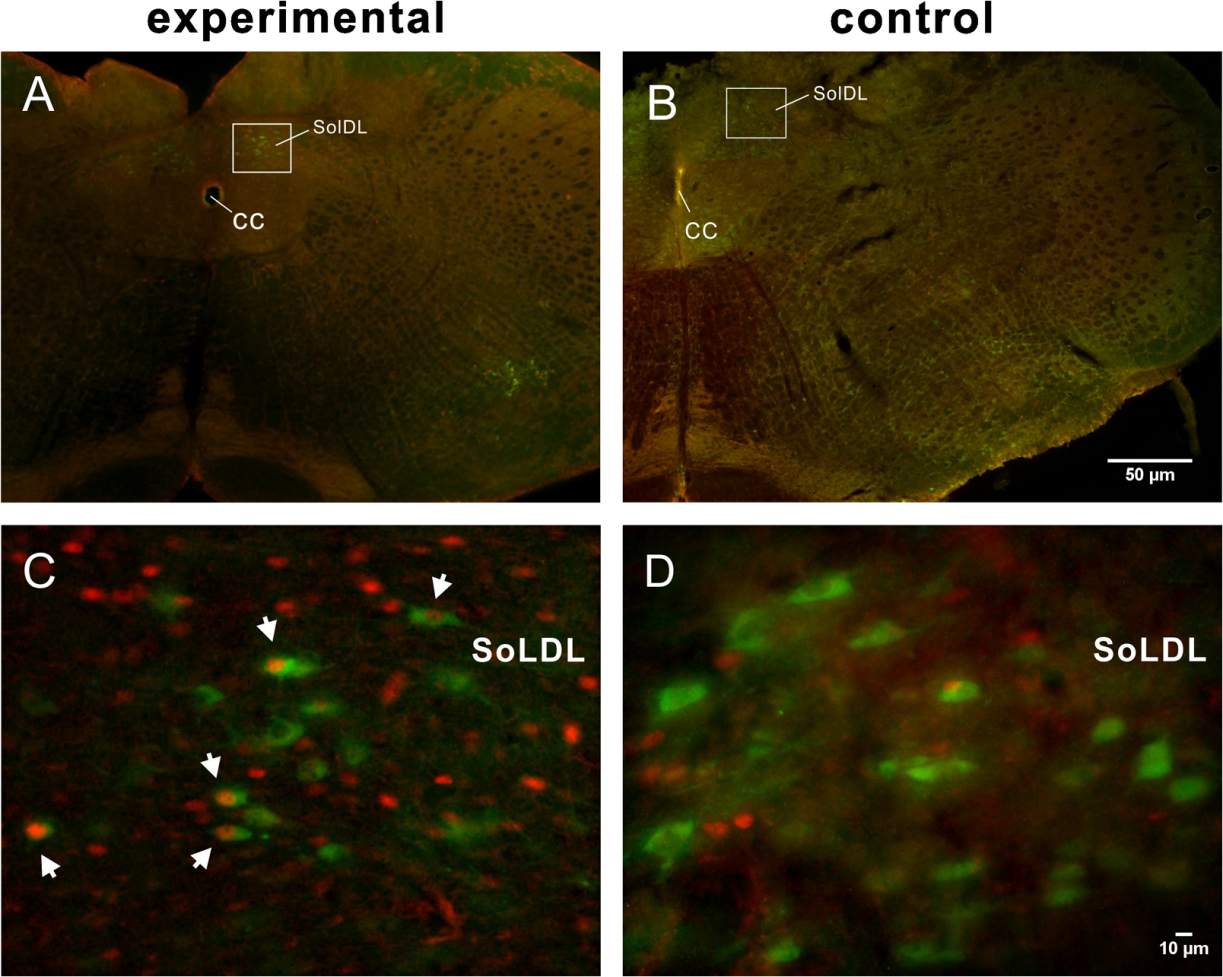
Low-power micrographs of the caudal medulla showing dual-labeled FLI and TH-ir neurons from experimental (A) and control (B) rats at the level of 0.8 mm caudal to the obex. Dorsal boxed areas in A and B, indicating the SolDL, are enlarged in C and D, respectively, showing double-labeled neurons (white arrows) in the experimental rat but no double labeling in the control rat. Scale bar = 50 μm (applies to A, B), 10 μm (applies to C, D).

**Fig 6:**
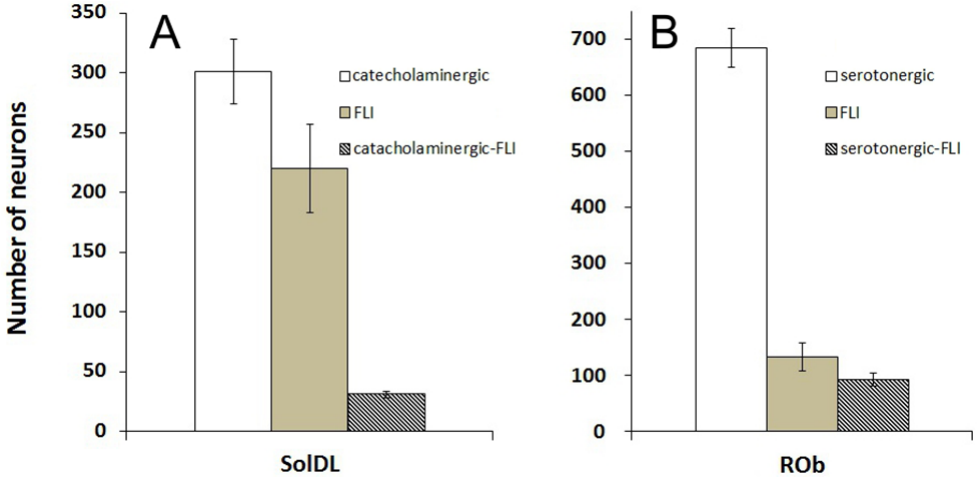
(A) The numbers of catecholaminergic neurons, FLI neurons and catecholaminergic FLI neurons in the SolDL activated by the LCR; (B) The numbers of serotonergic neurons, FLI neurons and serotoninergic FLI neurons in the ROb activated by the LCR.

In the caudal medulla, TH-ir neurons were mainly observed in the AP, the NTS, the dorsal motor nucleus of the vagus, and the ventrolateral medulla [24]. In the experimental group, the neurons showing both TH-ir and FLI were located in the bilateral SolDL at the level of 0.8 mm to 0.2 mm caudal to the obex (Fig 5C). The bilateral numbers of catecholaminergic neurons, FLI neurons and catecholaminergic FLI neurons in the SolDL were 301 ± 27, 220 ± 37 and 31 ± 3 respectively (n = 5, Fig 6A). Further analysis indicated that 12.71% ± 4.32% of the FLI neurons were TH-positive. No double-labeled neurons were observed in the NTS in the control group (Fig 5D). In the ventrolateral medulla, catecholaminergic FLI neurons in A1 showed similar distributions in the experimental and control groups (two-sample t-test, n = 5, *p* > 0.05).

### Distribution of serotoninergic-FLI neurons

**Fig 7:**
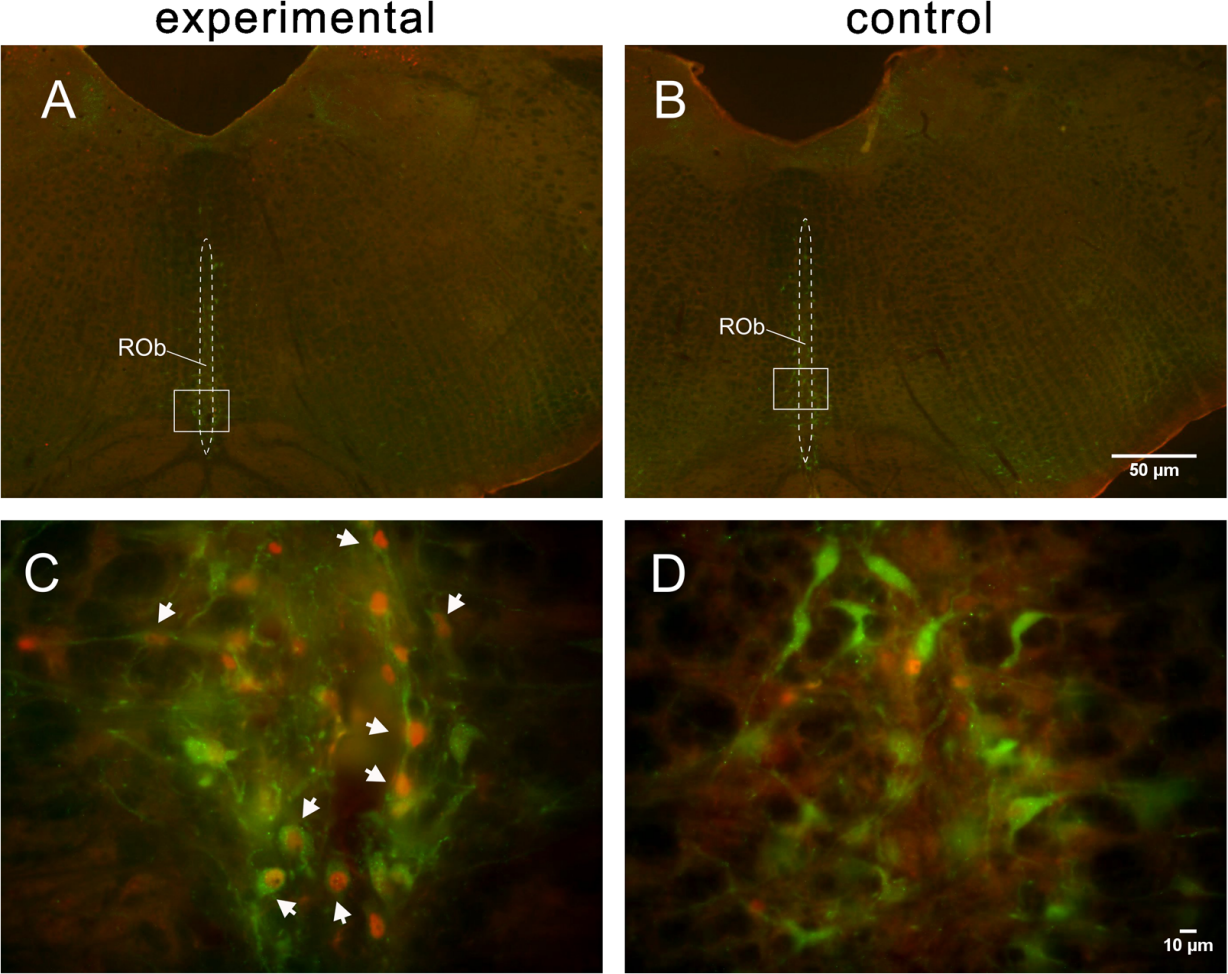
Low-power micrographs of the medulla with dual-labeled FLI and serotonin-ir neurons from experimental (A) and control (B) rats at the level of 0.6 mm rostral to the obex. Boxed areas in A and B, indicating the ROb, are enlarged in C and D, respectively, showing double-labeled neurons (white arrows) in the experimental rat but no double labeling in the control rat. Scale bar = 50 μm (applies to A, B), 10 μm (applies to C, D).

Serotoninergic neurons are mainly distributed in the raphe and its adjacent regions [25]. In the experimental group, neurons with intense FLI (191 ± 19, n = 5) were distributed primarily in the ROb (Fig 7C), between the obex and 1.2 mm rostral to the obex. The numbers of serotonergic neurons, FLI neurons and serotonergic FLI neurons in the ROb were 684 ± 35, 191 ± 19 and 93 ± 12 respectively (n = 5, Fig 6B). Further analysis indicated that 70.87% ± 13.20% of the FLI neurons were serotonin positive. Serotoninergic FLI neurons were also detected in the LPN from both groups, with no significant differences.

## Discussion

In the current study, we present the first description of the distribution of the medullary nuclei activated by the LCR in rats, indicating the potential central pathway of LCR in the medulla. Furthermore, we show for the first time that a number of the catecholaminergic neurons of the SolDL and of the serotoninergic neurons of the ROb were activated by the LCR. Additionally, a more practical surgical approach was developed for eliciting LCR in rats.

### Medullary nuclei involved in the LCR

Here, we describe the medullary distribution of neurons activated by the LCR in the bilateral NTS, VNC, AmbL, RVRG, Pre-Böt, Böt, VLR, SP5, and ROb. The data of control rats are consistent with previous studies that conducted in anesthetized animals together with surgical control [13,35]. The repeated exposure to the LCR resulted in strong intensity of FLI expression without influencing its specificity. Except for the NTS and AmbL, there have been few reports about these nuclei in the LCR pathway. Our data extended the knowledge on medullary nuclei that were involved in the LCR. However, previous investigations provided reliable evidence that FLI distributed in the NTS, VNC, nucleus ambiguus, RAmb, RVRG, VLR, SP5, AP, motor nucleus of vagus, inferior olive formation, lateral reticular formation and other medullary nuclei was involved in the central projection of laryngeal activity [13,35–37], which made our findings explicable.

#### Dorsal medulla

The afferent terminals of the SLN are mainly located in the one-third caudal of the NTS [38], which projects in part to the AmbL [8–10]. Focal warming and blocking of the adenosine A_2A_ receptor in the NTS has been shown to prolong apnea associated with the LCR, indicating that the NTS contributes to the LCR [39,40]. Without controversy that the NTS participates in the LCR, we further identified the subnuclei involved in this process. Except that FLI were only detected in the SolM and SolIM of experimental rats, the other subnuclei (sol, SolDL, SolI, SolV, SolVL, SolC) expressed more FLI than in controls. These subnuclei of the NTS have also been reported to be involved in upper respiratory activities [35–37]. Afferents from the SLN terminated in the SolI, SolC, SolM, SolIM, SolVL and SolDL [38,41,42]. FLI neurons in the sol, SolI, SolM, SolC and SolVL were activated in the laryngeal adductor response that is controlled by the lateral cricoarytenoid and thyroarytenoid muscles, which are also involved in the LCR [13]. Therefore, the subnuclei of NTS we described transmit information directly or indirectly to the LMN during the regulation of the LCR.

The medullary VNC (MVe, MVePC, MVeMC, SpVe, Pr) and SP5 (Sp5C, Sp5I, Pa5, sp5) showed abundant Fos expression in the present study. Megirian D. illustrated the vestibular control of LMN in cats [43]. Marina S. found that the medial and inferior vestibular nuclei were essential for the activity of the laryngeal muscles [44]. Because substantial levels of FLI were detected in the control group, the VNC nuclei also likely related to stress responses during anesthetization and surgery. Vestibulo-sympathetic system is critical to the compensatory autonomic adjustments in posture and movement [45]. Surgical operation in anaesthetic animals led changes in blood pressure as well as heart rate, which brought the outcome of large FLI in the VNC of controls [46,47]. When the LCR was elicited, stronger stress responses and reflex responses caused more FLI to express in experimental rats.

Laryngeal projections toward the SP5 have been reported in various species other than rats [12,38,48,49]. However, fos expression has been shown in the SP5 during coughing [35], the laryngeal adductor response [13] and tactile stimulation of the laryngeal vestibulum [37], which was consistent with that in our study. However, except for the association of SP5 with nociception and a few reports about its innervation of the laryngeal arch, the role of SP5 in the LCR pathway remains unknown.

#### Ventral medulla

During eupnea, laryngeal activity is generated by the respiratory network in the ventrolateral medulla [11,50,51]. During non-respiratory activities, respiratory neurons also influence the firing patterns of the LMN [50,52]. Our study shows that FLI was expressed in the RVRG, PrBöt and Böt during the LCR, indicating that a subset of respiratory neurons may mediate the LCR. However, the roles of RVRG, PrBöt and Böt neurons in respiratory or non-respiratory activity in the LCR require further study.

Neurons in the VLR (CVL, RVL) are mixed with C1 adrenaline cells, but are not themselves catecholamine containing [53]. In our study, several FLI neurons scattered in these regions did not show TH-ir double labeling, and those neurons were respiration-related and/or had cardiovascular function [53]. Transganglionic tracing of the SLN suggested that the VLR and reticular formation around Amb were descending nuclei from the NTS and were responsible for the upper respiratory actions [54]. Meanwhile, the apnea induced by the LCR was prolonged by inhibiting the extended rostral part of the VLR [6]. It can be concluded that certain functions of the VLR may be associated with respiratory activity of the LCR.

#### The Rob

Numerous neurons showing FLI were detected in the ROb. Holtman et al. found that activity in the RLN and phrenic motoneurons could be evoked by stimulation of the ROb [26], indicating that the ROb likely plays a role in the control of laryngeal activity. Verner et al. detected respiration depression by chemically stimulating the midline medulla [55] and further identified the innervation between medullary raphe nuclei and respiratory-related region [56], indicating that FLI of ROb likely relate to respiratory disturbance caused by the LCR. However, few studies have reported direct projections between the ROb and the nucleus ambiguus, and the function of the ROb within the laryngeal arch remains unclear.

### Medullary catecholaminergic neurons of the SolDL are involved in the LCR

In our study, catecholaminergic FLI neurons were detected in the SolDL at the level of 0.8 mm to 0.2 mm caudal to the obex, indicating that these catecholaminergic neurons contribute to the LCR. This finding is consistent with the pathological studies showing that catecholamines may be responsible for SIDS [27–29]. Our former data show that retrogradely labeled neurons from the caudal nucleus ambiguous are located in the SolDL and SolIM, and some of the retrogradely labeled neurons in the SolDL also expressed TH-ir at the level of 0.6 mm to 0.2 mm caudal to the obex [57], consistent with our present results, further indicating that SolDL catecholaminergic neurons may send direct projections to the LMN during the LCR. However, this hypothesis requires further study for confirmation.

### Medullary serotoninergic neurons of the ROb are involved in the LCR

The raphe system is involved in the pathophysiology of sleep apnea [58] and SIDS [30]. We demonstrate here that there are FLI serotoninergic neurons in the ROb between the obex and 1.2 mm rostral to the obex, indicating that a number of the ROb serotoninergic neurons contribute to the LCR. This finding is closely related to the pathological results showing that abnormalities in the serotonergic system are responsible for SIDS [30]. Further pharmacological study with administration of a serotonin antagonist in cats revealed that the excitation of the recurrent laryngeal nerves is likely mediated by serotonergic neurotransmitters originating from the ROb [26]. Therefore, consistent with previous reports, our study suggests that serotoninergic neurons of the ROb may act as pre-motoneurons in the LCR. Furthermore, we confirm that the serotoninergic terminals located among the LMN [21] originate at least in part from these double-labeled neurons in the ROb.

### Methodology

Researchers have previously elicited the LCR by dropwise stimulation of the laryngeal lumen of adults and neonates of various species [2,31,33]. However, dropwise stimulation is too weak to trigger sufficient expression of Fos for detection by immunofluorescence, and the diffusion of stimuli may evoke other reflexes that cause unrelated FLI. Therefore, infusion of KCl-HCl solution, which is thought to be the most effective stimulus for mucosa [20], was adopted in this study. Repeated stimulation enhanced the intensity of FLI than one single simulation, and the fixed protocol minimized the variation that was caused by operation. Respiratory disturbance and laryngeal muscle activities are the main responses of the LCR. Xia et al. characterized the elicitation of the LCR by monitoring respiratory disturbances reflected via the EMG of trunk musculature [31,59]. Similarly, we recorded the MTD of the same musculature, which successfully reflected the respiratory activity. The stronger stimulation and convincing respiratory disturbance (Fig 1B) confirm the elicitation of the LCR in our study.

In addition, we also observed the alterations of respiration during the repeated elicitation of the LCR. Hypertonic potassium and chloride to the receptor membrane effectively initiated the LCR [34]. The concentration of two ions had trend to reach balance in and out of the receptor membrane, meaning that the effect of stimulus was reduced during repeated infusion [7,33,34], which gave reasonable explanation for the results. Prolonged apnea generally occurs in neonates during the LCR, while adult rats were used in the study, showing short-time respiratory disturbance. Previous studies revealed that severe apnea did not always exhibited in adult animals during the LCR [7], indicating that mature development of brain facilitates central modulation against lethal responses induced by the LCR [60]. On the other hand, apnea may or may not appear depending on the type and strength of the stimulation [32].

Previous studies indicated that some medullary nuclei were potentially involved in the LCR [14,40,61–63]. Tracing studies revealed the laryngeal pre-motor neurons [14,61,62], showing overlaps with our finding. Pharmaceutical and functional approaches even provided the role of NTS in the LCR [40,63]. However, few reports identified the medullary nuclei under the LCR eliciting condition. In comparison, our study is more directly and precisely showing the distribution of the medullary nuclei activated by the LCR by using of FLI.

Fos protein is detected at low level under basal conditions, increases under stress, or fails to elicit under some behaviors, such as suckling [64]. Furthermore, analgesics and anesthetics also influence Fos protein expression [65]. The maximum of expression occurs between 1 and 3 h, then gradually disappears by 4–6 h after stimulation [66]. So animals were left unconsciously for 4 h after surgery to minimize the Fos expression caused by surgery or any other uncertain factors, and keep with anesthesia for 1 h to allow the FLI to reach the highest. However, the baseline of Fos expression and that elicited by anesthetics are unlikely to be avoided, which can be the reason of false positive FLI in the experimental group. Statistical analysis was carried out between experimental and control group to minimize the inevitable false positive FLI.

In summary, our study provides a better understanding of the central innervation pathways associated with the LCR and new neurobiological information about the catecholaminergic and serotoninergic neurons that contribute to the LCR.
